# Comorbidity Burden and Acute-Care Utilization in Adult Trauma Patients Across the Injury Severity Spectrum in a Nationwide Community-Based Survey (Korea, 2019–2023)

**DOI:** 10.3390/healthcare14101380

**Published:** 2026-05-18

**Authors:** Su-il Kim, Sung Mo Moon, Gwang-Seok Kim, Sung-Soo Choi, Min-Seok Choi, Jae-Seong Park, In-Hye Kang, Duk-Hee Lee, Yun-Deok Jang

**Affiliations:** 1 Department of Paramedicine, Yeungjin University, Daegu 41527, Republic of Korea; avantetop@hanmail.net; 2 Department of Paramedicine, Gwangju University, 277 Hyodeok-ro, Nam-gu, Gwangju 61743, Republic of Korea; smmoon82@hanmail.net (S.M.M.); emt515@naver.com (G.-S.K.); ranger898@naver.com (S.-S.C.); 3 Department of Health Administration, Yeungnam University College, Daegu 42415, Republic of Korea; dreebok@naver.com; 4 Department of Paramedicine, Busan Health University, Sari-ro 55 beon-gil, Saha-gu, Busan 49318, Republic of Korea; coma000@naver.com; 5 Department of Paramedicine, Daewon University, 316 Daehak-ro, Jecheon-si 27135, Republic of Korea; emtkih@daewon.ac.kr; 6 Department of Preventive Medicine, School of Medicine, Kyungpook National University, 680 Gukchaebosang-ro, Jung-gu, Daegu 41944, Republic of Korea; lee_dh@knu.ac.kr; 7 Department of Paramedicine, Tongmyong University, Busan 48520, Republic of Korea

**Keywords:** comorbidity, emergency department, hospitalization, injury severity score, length of stay, mortality, trauma, trauma registry

## Abstract

**Background:** This study aimed to evaluate the association between comorbidity and hospital admission, hospital length of stay (LOS), and in-hospital mortality among adult trauma patients across the injury severity spectrum in South Korea, and to assess whether these associations vary according to injury severity. **Methods**: We conducted a retrospective cohort study using the national Community-Based Severe Trauma Survey (2019–2023). Adult patients (≥18 years) with trauma were included after excluding records with missing key exposure or outcome variables. Comorbidity was defined using the ICD-10–based Elixhauser comorbidity framework. In addition to a binary classification (any vs. none), comorbidity burden was categorized into 0, 1, 2, and ≥3 conditions to evaluate dose–response relationships. The primary outcomes were hospital admission, LOS, and in-hospital mortality. Multivariable logistic regression models were used for admission and mortality, and regression models were applied for LOS, adjusting for demographic characteristics, injury mechanism, physiologic status, and system-level factors. Effect modification by injury severity was assessed using interaction terms and ISS-stratified analyses. **Results:** Among 49,259 patients, 32,999 (67.0%) had at least one comorbidity. Patients with comorbidities were older, had higher injury severity, and showed higher admission rates, longer LOS, and higher in-hospital mortality compared with those without comorbidities. After adjustment, comorbidity remained independently associated with increased odds of admission, prolonged LOS, and in-hospital mortality. A dose–response relationship was observed, with increasing comorbidity burden associated with progressively worse outcomes (*p* for trend < 0.001). In addition, substantial heterogeneity was identified across individual comorbidities, with conditions such as metastatic cancer, liver disease, coagulopathy, renal disease, and fluid and electrolyte disorders showing stronger associations with adverse outcomes. The magnitude of these associations varied across ISS strata, indicating injury severity-dependent effects. **Conclusions:** In this nationwide cohort, comorbidity burden and type were important determinants of acute-care utilization and in-hospital mortality among trauma patients. Incorporating comorbidity information into early risk stratification may improve prognostic accuracy and support more efficient resource allocation and clinical decision-making across the trauma care continuum.

## 1. Introduction

Trauma is a leading cause of death and disability worldwide, and even among patients who survive the prehospital phase, it is highly likely to be accompanied by substantial resource utilization and poor outcomes during the acute-care trajectory from the emergency department to surgery or interventional procedures and subsequent intensive care [1,2]. Factors known to influence clinical outcomes in trauma patients include injury mechanism, injury severity, hemorrhage and physiological instability, time to hospital arrival, and institutional capacity and trauma system characteristics [2].

However, these traditional prognostic factors alone are insufficient to fully explain the heterogeneity observed among patients with similar injury severity in terms of admission needs, length of stay (LOS), and in-hospital mortality [3,4]. Meanwhile, with population aging and the increasing prevalence of chronic diseases, the proportion of trauma patients with comorbidities continues to rise [5].

Comorbidities may reduce physiological reserve after trauma, increasing the risk of complications, and may also affect diagnostic and therapeutic pathways and treatment intensity through constraints such as the use of anticoagulants or antiplatelet agents and limitations in cardiopulmonary function [6]. Consequently, comorbidities may influence admission decisions, recovery trajectories, and discharge planning both directly and indirectly, thereby contributing to prolonged LOS and a higher risk of in-hospital death [3,4,7].

Moreover, the impact of comorbidities may extend beyond a simple presence/absence effect, varying by comorbidity type and cumulative burden, and may differ according to injury severity [6,8].

Prior studies have reported that comorbidities are associated with mortality, complications, functional recovery, and healthcare resource utilization among trauma patients [6,7]. Some investigations have evaluated overall risk using comorbidity indices such as the Charlson Comorbidity Index or the Elixhauser comorbidity measure, while others have examined how specific chronic conditions affect post-trauma outcomes [6,8,9]. Nevertheless, the existing evidence has several limitations. First, many studies rely on single-center or hospital-based data, which may limit generalizability [7].

In addition, by analyzing trauma patients as a whole, some studies do not sufficiently disentangle findings specific to Trauma patients, and relatively few have evaluated admission, LOS, and in-hospital mortality simultaneously within a single analytic framework, as many have focused primarily on mortality or complications [6,7]. Furthermore, although the effects of comorbidities may vary by injury severity, evidence that systematically examines effect modification using severity stratification or interaction approaches remains limited [6,8]. In South Korea, despite the growing number of large database studies, further evidence is needed from Community-Based Severe Trauma Survey data that focuses on trauma patients, evaluates the impact of comorbidities across multiple outcomes (admission, LOS, and in-hospital mortality), and explores differences according to injury severity [10,11].

Therefore, the aim of this study is to analyze the association between comorbidities and admission, LOS, and in-hospital mortality among patients with among adults with Trauma patients across severity spectrum using a Community-Based Severe Trauma Survey dataset in South Korea from 2019 to 2023. By leveraging community-based data to enhance generalizability and by adopting multiple outcomes that capture both healthcare utilization and prognosis, this study seeks to broaden the clinical interpretation of comorbidity burden. In addition, by exploring effect differences across injury severity levels, our findings may provide evidence to support early risk stratification and resource allocation strategies for trauma patients.

Therefore, the specific aims of this study were:

(i) To determine the prevalence of comorbidities among trauma patients;
(ii) To evaluate the association between comorbidity and hospital admission, length of stay, and in-hospital mortality;
(iii) To assess whether these associations differ according to injury severity.

## 2. Materials and Methods

### 2.1. Study Design and Data Source

This study is a retrospective cohort analysis using the Community-Based Severe Trauma Survey database in South Korea. The survey is an official statistical investigation designed to generate regional- and facility-level estimates of the occurrence, care processes, and clinical outcomes of Trauma patients and mass-casualty events, and is conducted under strict confidentiality protections. Data are collected using a standardized medical record abstraction instrument ‘Community-Based Trauma patients Mass-Casualty Medical Record Survey Form’ completed by trained survey personnel. The study period (2019–2023) was selected to reflect the most recent and complete nationwide dataset available at the time of analysis.

The data collection process follows a structured workflow consisting of case identification and standardized chart review. First, eligible cases are identified from a pre-specified case list using the EMS run-sheet serial number. Surveyors then document the receiving hospital region and hospital code/name and confirm whether the medical record is accessible; when records are not obtainable, the reason is recorded. Using the standardized survey form, trained abstractors systematically extract patient- and event-level variables from EMS documentation and hospital charts, including demographics, injury circumstances, initial physiological status, injury severity measures, key interventions, disposition, length of stay, and in-hospital outcomes, thereby ensuring consistent variable definitions across participating sites. The Community-Based Severe Trauma Survey dataset used in this study was obtained through a formal data request to the data-holding authority responsible for the survey (via an institutional application and data-use agreement). Access is provided only for approved research purposes, and the dataset is released in a de-identified format; investigators are required to comply with data security and confidentiality policies stipulated by the provider. This study used de-identified secondary data, and the research protocol received Institutional Review Board (IRB) exemption from the authors’ affiliated institution in accordance with applicable regulations and institutional policies for research using anonymized data. Ethical review was waived because the study used fully de-identified secondary data and posed minimal risk to participants. This study was approved by the Institutional Review Board of Kyungpook National University (IRB No.: 2025-0322).

### 2.2. Study Population

**Figure 1:**
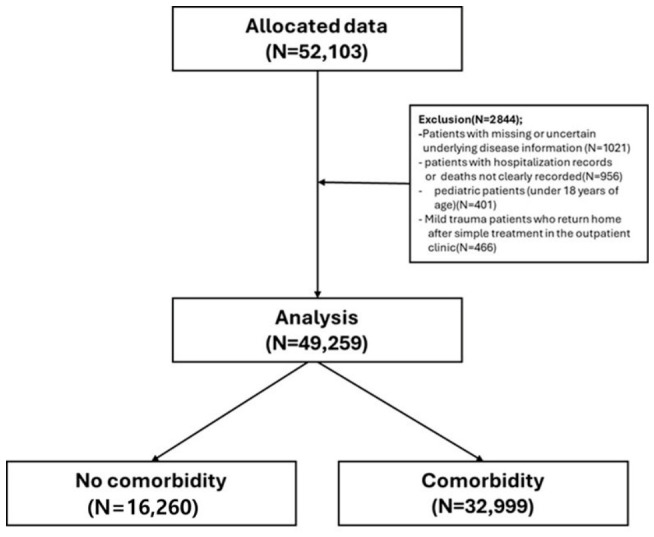
Study flow diagram.

We identified 52,103 trauma cases from the Community-Based Severe Trauma Survey database between 1 January 2019 and 31 December 2023, including patients who presented to participating emergency medical facilities. After applying predefined eligibility and data-quality criteria, we excluded 2844 cases for the following reasons: missing or uncertain comorbidity information (*n* = 1021), hospitalization status or death outcomes not clearly recorded (*n* = 956), pediatric patients younger than 18 years (*n* = 401), and mild trauma cases discharged home after simple outpatient treatment (*n* = 466). After these exclusions, the final analytic cohort comprised 49,259 patients. The cohort was then categorized according to comorbidity status into a no-comorbidity group (*n* = 16,260) and a comorbidity group (*n* = 32,999), as shown in the study flow diagram. (Figure 1). A complete-case analysis was performed by excluding cases with missing key variables, which may introduce potential selection bias. To assess potential selection bias due to exclusion of cases with missing data, baseline characteristics between included and excluded patients were compared.

### 2.3. Exposure, Outcomes, and Covariates

The primary exposure was comorbidity. Comorbidities were identified using the ICD-10–based Elixhauser comorbidity framework and operationalized as the presence of any comorbidity (yes/no). Comorbidities were identified using the ICD-10 coding algorithm validated by Quan et al., based on the original Elixhauser methodology [2]. In additional analyses, comorbidity burden was further characterized using the number of Elixhauser conditions and, where appropriate, individual comorbidity indicators (present/absent) for clinically relevant chronic diseases. Comorbidity burden was additionally categorized into 0, 1, 2, and ≥3 conditions to assess potential dose–response relationships across outcomes.

The primary outcomes were hospital admission, hospital length of stay (LOS), and in-hospital mortality. Admission was defined as admission to the hospital following the emergency department visit (yes/no). LOS was primarily analyzed as a binary outcome (prolonged hospitalization, ≥7 days) to avoid potential misinterpretation due to early mortality or minor injuries. As a secondary utilization metric, prolonged hospitalization was defined as LOS ≥ 7 days.

Covariates were selected a priori based on clinical relevance and prior trauma literature to reduce confounding. These included demographic characteristics (age and sex), injury-related factors (injury mechanism and injury severity), and clinical status at presentation. Injury severity was captured using the Injury Severity Score (ISS), and baseline neurological status was assessed using the Glasgow Coma Scale (GCS) when available. Physiological variables obtained at emergency department presentation included systolic blood pressure and heart rate, and additional vital signs (respiratory rate, oxygen saturation, and temperature) were incorporated when recorded. We also accounted for system-level and care-process factors, including the level/type of receiving facility (trauma center and emergency center categories), transfer status and/or transport mode where available, and time-related variables such as arrival time categories (daytime vs. nighttime; weekday vs. weekend) when recorded in the survey. For LOS-focused models, we additionally considered treatment intensity and care pathway indicators available in the dataset, such as operative intervention (yes/no) and intensive care unit (ICU) admission, to improve model fit and interpretability in sensitivity analyses. Comorbidity was additionally categorized into clinically meaningful groups based on organ systems (e.g., cardiovascular, metabolic, psychiatric, renal) following previous literature. Prolonged hospitalization was defined as LOS ≥ 7 days based on prior trauma studies and to distinguish patients requiring extended acute care from short-stay or rapidly discharged patients.

### 2.4. Statistical Analysis

Statistical analyses were conducted using IBM SPSS Statistics, Version 27.0 (IBM Corp., Armonk, NY, USA) and MedCalc Statistical Software, Version 20 (MedCalc Software Ltd., Ostend, Belgium), in accordance with the pre-specified analytic plan. Baseline characteristics were summarized by comorbidity status. Categorical variables are presented as frequencies and percentages and were compared using the chi-square test (or Fisher’s exact test when expected cell counts were small). Continuous variables were evaluated for distributional characteristics and summarized as mean ± standard deviation when approximately normally distributed or as median (interquartile range) otherwise; between-group comparisons used the independent-samples *t*-test for approximately normal variables and the Mann–Whitney U test for non-normally distributed variables.

For the primary outcome (hospital admission; yes/no), we fitted multivariable logistic regression models to estimate adjusted odds ratios (ORs) with 95% confidence intervals (CIs) for the association between comorbidity and admission after covariate adjustment. For the secondary outcome (in-hospital mortality; yes/no), we similarly applied multivariable logistic regression to estimate adjusted ORs and 95% CIs. For the tertiary outcome (hospital length of stay, days). For LOS, multivariable logistic regression models were used to estimate the association between comorbidity and prolonged hospitalization (≥7 days). The cutoff of ≥7 days was selected based on previous trauma studies and clinical relevance to distinguish short-term hospitalization from prolonged resource utilization. Patients who died before the threshold were classified as having prolonged hospitalization. Because LOS is typically right-skewed, we assessed model assumptions and conducted sensitivity analyses using alternative specifications when warranted (log-transformed LOS or generalized linear models with appropriate link and variance functions). Normality of continuous variables was assessed using the Kolmogorov–Smirnov test, and homogeneity of variance was evaluated using Levene’s test.

To evaluate potential effect modification by injury severity, we performed stratified analyses across ISS categories (1–8, 9–15, 16–24, and ≥25). Within each ISS stratum, admission and in-hospital mortality were analyzed using adjusted logistic regression models, and LOS was analyzed using adjusted regression models, applying the same covariate adjustment strategy. For comorbidity burden categories (0, 1, 2, and ≥3), *p* for trend was calculated using the linear-by-linear association test for categorical outcomes and linear regression for continuous outcomes. All statistical tests were two-sided, and *p*-values < 0.05 were considered statistically significant.

## 3. Results

### 3.1. General Characteristics

The results of the analysis of general characteristics are presented in Table 1. A total of 49,259 patients were included, with a mean age of 48 (30–66) years. Male patients accounted for 30,162 (60.3%) and female patients for 19,097 (39.7%). Most patients resided in urban areas (33,850; 68.7%), while 15,409 (31.3%) lived in rural (non-urban/provincial) areas. Regarding the type of emergency department visited, local emergency medical centers were the most common (18,500; 37.6%), followed by regional emergency medical centers (14,000; 28.4%), local emergency medical facilities (12,000; 24.4%), and regional trauma centers (4759; 9.7%). Trauma events occurred most frequently in the afternoon (12:00–17:59; 15,259; 31.0%) and evening (18:00–23:59; 15,500; 31.5%), followed by the morning (06:00–11:59; 12,300; 25.0%), with the lowest frequency at night (00:00–05:59; 6200; 12.6%).

The most common mechanism of injury was traffic accidents (21,166; 43.0%), followed by falls (14,777; 30.0%), penetrating injuries (3448; 7.0%), and other mechanisms (9868; 20.0%). For clinical variables at presentation, the mean systolic blood pressure was 112 ± 22.0 mmHg, the mean heart rate was 109 ± 26.0 bpm, and the mean respiratory rate was 13.0 ± 7.01 /min. The median Glasgow Coma Scale score was 14 (IQR 13–15). Regarding discharge outcomes assessed using the Glasgow Outcome Scale, good recovery was observed in 37,829 (76.8%), moderate disability in 6900 (14.0%), severe disability in 2960 (6.0%), vegetative state in 520 (1.1%), and death in 1118 (2.3%). For the study outcomes, the admission rate was 38,428 (78.0%), in-hospital mortality occurred in 1118 (2.2%), and the median hospital length of stay was 32 days (IQR 2–60) (Table 1). Baseline characteristics of included and excluded patients were compared, and no substantial differences were observed in key demographic variables. The relatively long median LOS observed in this study may reflect the inclusion of extended hospitalization periods, including post-acute care and rehabilitation, as well as system-level factors such as inter-facility transfer delays and bed availability. To assess potential selection bias due to exclusion of cases with missing data, baseline characteristics between included and excluded patients were compared. GOS-defined death and in-hospital mortality were consistent across analyses (Table 1).

The results of the analysis of comorbidity characteristics are presented in Table 2. Among the total study population, 16,260 (33.0%) patients had no comorbidity, whereas 32,999 (67.0%) had ≥1 comorbidity. Regarding the distribution of comorbidity types, congestive heart failure was the most common, with 8629 (26.1%) cases, followed by cardiac arrhythmia with 7657 (23.2%), psychosis with 4247 (12.9%), and renal disease with 3142 (9.5%). Other comorbidities included hypertension with 1402 (4.2%), peripheral vascular disease with 1232 (3.7%), diabetes mellitus with 1021 (3.1%), valvular disease with 899 (2.7%), pulmonary circulation disorders with 851 (2.6%), chronic pulmonary disease with 625 (1.9%), and paralysis with 608 (1.8%). Less frequent conditions were fluid and electrolyte disorders with 330 (1.0%), anemia (blood loss/deficiency) with 320 (1.0%), solid tumor without metastasis with 310 (0.9%), obesity with 300 (0.9%), liver disease with 260 (0.8%), other neurological disorders with 210 (0.6%), peptic ulcer disease with 200 (0.6%), depression with 186 (0.6%), alcohol abuse with 180 (0.5%), metastatic cancer with 140 (0.4%), and drug abuse with 80 (0.2%) (Table 2).

### 3.2. Comorbidity Burden and Its Association with Clinical Outcomes

Comorbidity burden was significantly associated with adverse clinical outcomes among trauma patients (Table 3). Admission rates, in-hospital mortality, and length of hospital stay (LOS) increased progressively with a greater number of comorbid conditions. Patients without comorbidity had an admission rate of 73.3% and an in-hospital mortality of 1.8%, whereas those with ≥3 comorbidities showed substantially higher admission (82.8%) and mortality rates (3.2%). A clear dose–response relationship was observed between comorbidity burden and mortality risk. Compared with patients without comorbidity, those with one comorbid condition had a modestly increased risk of mortality (adjusted odds ratio [aOR] 1.29, 95% confidence interval [CI] 1.08–1.55). This risk further increased in patients with two comorbidities (aOR 1.55, 95% CI 1.30–1.85) and was highest in those with three or more comorbidities (aOR 1.92, 95% CI 1.58–2.33), with a statistically significant trend across groups (*p* for trend < 0.001).

Post hoc pairwise comparisons confirmed that mortality risk was significantly higher in patients with two or more comorbidities compared with those without comorbidity (all *p* < 0.001), whereas the difference between patients with one comorbidity and those without comorbidity was modest but statistically significant.

Similarly, LOS increased in a stepwise manner with increasing comorbidity burden. The median LOS was 28 days (interquartile range [IQR] 2–58) in patients without comorbidity, compared with 31 days (IQR 2–60), 35 days (IQR 2–62), and 39 days (IQR 3–65) in patients with one, two, and ≥3 comorbidities, respectively.

Analysis of individual Elixhauser comorbidities demonstrated substantial heterogeneity in their associations with clinical outcomes. Several conditions, including metastatic cancer, liver disease, coagulopathy, renal disease, and fluid and electrolyte disorders, were strongly associated with increased mortality and prolonged LOS, whereas hypertension and hypothyroidism showed no significant association after adjustment (Table 4).

### 3.3. Comparison of Demographic Characteristics of Trauma Patients According to Comorbidity Status

The comparison of participant characteristics according to comorbidity status is presented in Table 5. The differences in age and injury severity (ISS) showed large (Cohen’s d = 0.75) and moderate (Cohen’s d = 0.45) effect sizes, respectively, while the association between comorbidity and hospital admission demonstrated a small-to-moderate effect size (Cramer’s V = 0.08). Patients with comorbidity were significantly older than those without comorbidity (61 (47–74) vs. 45 (28–62) years, *p* < 0.001), and the proportion of females was higher in the comorbidity group (12,527 [42.8%] vs. 6570 [38.1%], *p* < 0.001). The distribution of residential area differed between groups, with a higher proportion of rural residents and a lower proportion of urban residents in the comorbidity group (rural: 11,299 [32.2%] vs. 4110 [25.2%]; urban: 21,700 [67.8%] vs. 12,150 [74.8%], *p* = 0.01). Injury severity was also greater in the comorbidity group, as indicated by a higher ISS (10 [6–19] vs. 8 [4–15], *p* < 0.001).

In terms of outcomes, the comorbidity group had a higher admission rate (25,538 [79.8%] vs. 11,920 [73.3%], *p* < 0.001), higher in-hospital mortality (808 [2.5%] vs. 310 [1.8%], *p* < 0.001), and a longer hospital length of stay (36 [2–62] vs. 28 [2–58] days, *p* = 0.002). Glasgow Outcome Scale distributions also differed, with higher proportions of death (808 [2.5%] vs. 310 [1.8%], *p* < 0.001), vegetative state (380 [1.2%] vs. 140 [0.8%], *p* = 0.02), and severe disability (2040 [6.4%] vs. 920 [5.3%], *p* = 0.01) in the comorbidity group, whereas good recovery was less frequent (25,181 [75.6%] vs. 12,580 [78.7%], *p* = 0.001). The proportion of moderate disability did not differ significantly between groups (4590 [14.3%] vs. 2310 [13.4%], *p* = 0.10).

Regarding the time of trauma occurrence, injuries occurred more frequently in the afternoon in the comorbidity group (10,249 [32.0%] vs. 4010 [24.8%], *p* = 0.02), whereas evening occurrences were less frequent (9900 [30.9%] vs. 5600 [34.4%], *p* = 0.04). Injury mechanisms differed modestly between groups: traffic accidents were slightly more common in the comorbidity group (14,166 [44.3%] vs. 7000 [43.0%], *p* = 0.01), penetrating injuries were less common (3048 [6.4%] vs. 1400 [8.6%], *p* = 0.003), and other blunt/unknown injuries were more common (6188 [19.3%] vs. 2680 [16.6%], *p* = 0.02). The proportion of falls did not differ between groups (9597 [30.0%] vs. 5180 [31.8%], *p* = 0.92).

For additional clinical variables at presentation, the comorbidity group had lower systolic blood pressure (109 ± 23.00 vs. 116 ± 21.01 mmHg, *p* = 0.002) and a lower heart rate (104 ± 25 vs. 112 ± 27 bpm, *p* < 0.001). Respiratory rate did not differ significantly (12.9 ± 6.5 vs. 13.1 ± 7.8 /min, *p* = 0.219). Neurologic status was poorer in the comorbidity group, with a lower GCS (9 [5–14] vs. 13 [9–15], *p* = 0.001).

In prehospital characteristics, prehospital cardiac arrest was more frequent in the comorbidity group (3896 [12.2%] vs. 1820 [10.6%], *p* = 0.001), and the call-to-hospital arrival time was slightly longer (34 [23–49] vs. 32 [22–45] min, *p* = 0.04). Regarding transport method, ambulance (119) use was higher in the comorbidity group (25,343 [79.2%] vs. 13,100 [76.0%], *p* = 0.002), whereas personal vehicle use was lower (5757 [18.0%] vs. 3600 [20.9%], *p* = 0.001); helicopter/other transport did not differ significantly (899 [2.8%] vs. 530 [3.1%], *p* = 0.20) (Table 5).

**Figure 2:**
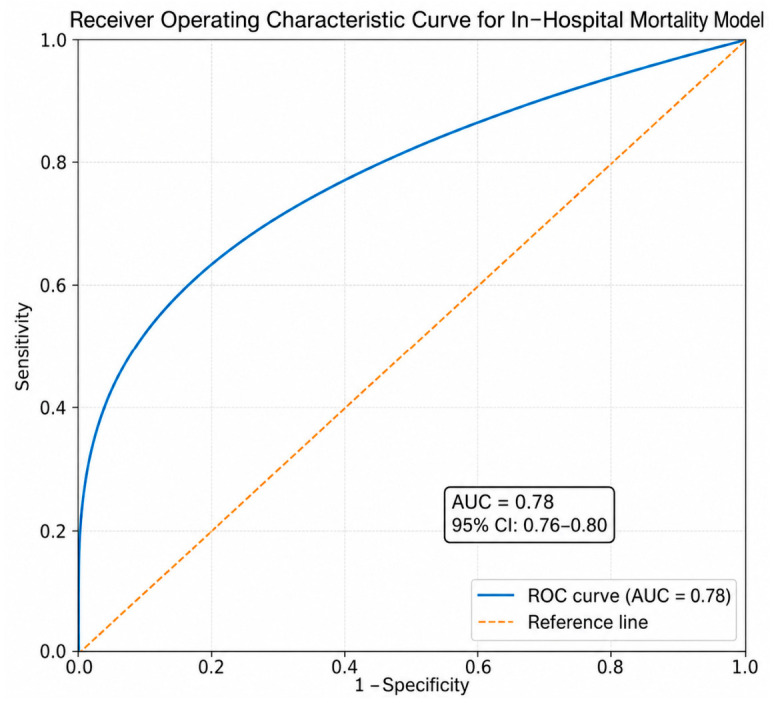
Receiver operating characteristic (ROC) curve for the in-hospital mortality model. The model demonstrated acceptable discrimination, with an area under the curve (AUC) of 0.78 (95% CI: 0.76–0.80).

The strength of these associations varied across ISS strata, indicating injury-severity–dependent effects. A dose–response relationship was observed, with increasing comorbidity burden associated with worse outcomes (*p* for trend < 0.001). The interaction between comorbidity and ISS was statistically significant (*p* for interaction = 0.01). The mortality prediction model demonstrated acceptable discrimination, with an AUC of 0.78 (95% CI: 0.76–0.80) (Figure 2).

### 3.4. Multivariable Regression Analysis of Admission, in-Hospital Mortality, and Prolonged Length of Stay

In multivariable logistic regression analysis, the presence of comorbidity was independently associated with increased odds of hospital admission (adjusted OR 1.32, 95% CI 1.25–1.39, *p* < 0.001). Similarly, comorbidity was significantly associated with higher in-hospital mortality (adjusted OR 1.41, 95% CI 1.22–1.63, *p* < 0.001). For prolonged hospitalization (length of stay ≥ 7 days), comorbidity was also identified as a significant predictor (adjusted OR 1.28, 95% CI 1.21–1.35, *p* < 0.001). Among covariates, increasing age was consistently associated with higher odds of admission (adjusted OR 1.02 per year, 95% CI 1.02–1.03, *p* < 0.001), in-hospital mortality (adjusted OR 1.03, 95% CI 1.02–1.04, *p* < 0.001), and prolonged hospitalization (adjusted OR 1.01, 95% CI 1.01–1.02, *p* < 0.001). Injury severity, as measured by ISS, was also significantly associated with all outcomes, with higher ISSs corresponding to increased odds of admission (adjusted OR 1.06, 95% CI 1.05–1.07, *p* < 0.001), mortality (adjusted OR 1.08, 95% CI 1.06–1.10, *p* < 0.001), and prolonged length of stay (adjusted OR 1.04, 95% CI 1.03–1.05, *p* < 0.001). Prehospital cardiac arrest was strongly associated with worse outcomes, including higher odds of admission (adjusted OR 2.15, 95% CI 1.98–2.33, *p* < 0.001), markedly increased in-hospital mortality (adjusted OR 3.82, 95% CI 3.21–4.55, *p* < 0.001), and prolonged hospitalization (adjusted OR 1.47, 95% CI 1.33–1.62, *p* < 0.001). Sex was not significantly associated with in-hospital mortality or prolonged hospitalization after adjustment, although female patients had slightly higher odds of hospital admission (adjusted OR 1.08, 95% CI 1.02–1.14, *p* = 0.006) (Table 6).

## 4. Discussion

This study used the Community-Based Severe Trauma Survey data in South Korea from 2019 to 2023 to evaluate the associations of comorbidity status with hospital admission, length of stay (LOS), and in-hospital mortality among trauma patients with varying injury severity, and to explore whether these associations differed according to injury severity (ISS), suggesting potential effect modification. The analysis showed that approximately two-thirds of the study population had at least one comorbidity, and compared with patients without comorbidity, those with comorbidity were older, had a higher proportion of females, and had greater injury severity (ISS) [10].

In addition, the comorbidity group had significantly higher admission and in-hospital mortality rates and a longer LOS; functional outcomes assessed by the Glasgow Outcome Scale (GOS) were also poorer, with higher proportions of death, vegetative state, and severe disability and a lower proportion of good recovery, suggesting that comorbidity is associated with less favorable healthcare utilization and outcomes in trauma patients [10].

Importantly, our study extends these findings by demonstrating a clear dose–response relationship between comorbidity burden and adverse clinical outcomes. As the number of comorbid conditions increased, admission rates, mortality, and LOS consistently worsened, with patients having ≥3 comorbidities showing the highest mortality risk. This finding suggests that comorbidity burden functions as a cumulative risk factor rather than a binary exposure and highlights the importance of quantifying comorbidity load in trauma prognosis [11].

These findings are generally consistent with prior studies reporting that comorbidity among trauma patients is associated with increased mortality, complications, poorer functional recovery, and greater healthcare resource utilization [11]. In particular, comorbidity burden may reduce physiological reserve during the acute phase, increasing the risk of complications such as hemorrhage, infection, and respiratory failure, and may complicate diagnostic, therapeutic, and surgical strategies due to factors such as anticoagulant or antiplatelet use and limitations in baseline cardiopulmonary and renal function, thereby increasing the likelihood of admission, prolonged hospitalization, and death [11].

Furthermore, our analysis of individual Elixhauser comorbidities revealed substantial heterogeneity in their impact on outcomes. Conditions such as metastatic cancer, liver disease, coagulopathy, renal disease, and fluid and electrolyte disorders were strongly associated with increased mortality and prolonged LOS, whereas others, including hypertension and hypothyroidism, showed no significant association after adjustment. These findings indicate that comorbidity should not be treated as a homogeneous construct and that condition-specific effects must be considered in trauma risk assessment [12].

The observation that the comorbidity group had higher rates of prehospital cardiac arrest, longer call-to-hospital arrival times, and greater use of ambulance transport (119) indicates that the influence of comorbidity may not be confined to in-hospital care but may extend across the entire care pathway, including vulnerability in the prehospital phase [11]. The higher rate of prehospital cardiac arrest observed in patients with comorbidities may be explained by reduced physiological reserve and a higher prevalence of underlying cardiovascular conditions such as heart failure and arrhythmia, which increase vulnerability to rapid clinical deterioration following traumatic injury [11].

Previous studies have quantified comorbidity using indices such as the Charlson or Elixhauser measures [12]. The Charlson Comorbidity Index has been widely used for mortality prediction, whereas the Elixhauser framework provides a broader representation of chronic disease burden and allows for more detailed evaluation of condition-specific heterogeneity [12,13].

By incorporating both comorbidity burden and individual Elixhauser conditions, our study enhances clinical interpretability beyond a simple “presence vs. absence” classification and provides a more nuanced understanding of how comorbidities influence trauma outcomes [13]. Prior evidence has often been limited by reliance on single-center or hospital-based data, which constrains generalizability, and by a focus on single outcomes such as mortality or complications [14].

In contrast, our study used a nationwide community-based dataset and simultaneously evaluated admission, LOS, and in-hospital mortality, thereby providing a more comprehensive understanding of how comorbidity influences both prognosis and healthcare utilization across the trauma care continuum [14]. The relatively long LOS observed in this study may reflect inclusion of prolonged hospitalization and post-acute care, such as rehabilitation, as well as system-level factors such as inter-facility transfer delays and resource availability [14].

The finding that the comorbidity group was older and had higher injury severity is also consistent with reports indicating that population aging and increasing chronic disease prevalence represent a growing burden on trauma systems [15]. Older trauma patients may have poorer physiological compensation even with similar injuries, and outcomes can worsen when combined with polytrauma and medication-related factors such as anticoagulant use, emphasizing the importance of comorbidity-informed risk stratification [15]. Moreover, the interaction between comorbidity and injury severity suggests that their combined effect may be synergistic rather than additive. Multiorgan functional impairment can complicate fluid resuscitation, surgical timing, and intensive care management, increasing the risk of complications and prolonged hospitalization [16].

Studies based on single-institution trauma registry data have also reported associations between comorbidity and mortality and resource utilization [17]. However, our findings derived from community-based survey data provide greater external validity and reflect real-world clinical variability across institutions [17].

From a policy perspective, these findings suggest that trauma system performance can be improved by incorporating comorbidity information into early risk stratification and resource allocation strategies [18]. Standardizing comorbidity data collection in prehospital settings and integrating it with injury severity may support more effective triage decisions, early ICU planning, and multidisciplinary care coordination [18,19,20].

Finally, this study has several limitations. First, as an observational study, causal inference is limited, and residual confounding may remain. Second, comorbidity data were derived from ICD-10 codes and may be subject to underreporting or misclassification. Third, although comorbidity burden was categorized to assess dose–response relationships, this approach does not capture disease severity or control status. Fourth, LOS may be influenced by system-level factors not fully captured in the dataset. Although comorbidity burden was categorized into 0, 1, 2, and ≥3 conditions, this count-based approach may not fully reflect the clinical heterogeneity or severity of individual diseases.

## 5. Conclusions

Using the Community-Based Severe Trauma Survey data in South Korea (2019–2023), this study demonstrated that comorbidity is highly prevalent among trauma patients and is significantly associated with increased hospital admission, prolonged length of stay (LOS), and higher in-hospital mortality. Importantly, the impact of comorbidity was not uniform; rather, a clear dose–response relationship was observed, with increasing comorbidity burden associated with progressively worse clinical outcomes.

In addition, substantial heterogeneity was identified across individual comorbid conditions. Certain comorbidities, including metastatic cancer, liver disease, coagulopathy, renal disease, and fluid and electrolyte disorders, were strongly associated with increased mortality and prolonged hospitalization, whereas others showed limited or no independent association after adjustment. These findings suggest that both the number and type of comorbid conditions should be considered in trauma risk assessment.

Overall, these results highlight the importance of incorporating structured comorbidity information into early risk stratification and clinical decision-making across the trauma care continuum, including prehospital triage, inter-facility transfer, and in-hospital resource allocation. Integrating comorbidity burden and condition-specific risk into trauma care pathways may improve prognostic accuracy and support more efficient use of healthcare resources.

Future research should focus on developing and validating comorbidity-informed prediction models and evaluating whether targeted multidisciplinary management strategies for high-risk comorbidity profiles can improve clinical outcomes and reduce variability in trauma care.

## Figures and Tables

**Table 1:** Baseline characteristics of patients (*N* = 49,259).

Variables	Trauma Patients (N = 49,259)
Age, years, median (IQR)	48 (30–66)
Male, n (%)	30,162 (60.3)
Female, n (%)	19,097 (39.7)
Residential area of patients	
Urban	33,850 (68.7)
Rural (Provinces, non-urban)	15,409 (31.3)
Type of ED visited, n (%)	
Local emergency medical facility	12,000 (24.4)
Local emergency medical center	18,500 (37.6)
Regional emergency medical center	14,000 (28.4)
Regional trauma center	4759 (9.7)
Time of trauma occurrence, n (%)	
Morning (from 06:00 to 11:59)	12,300 (25.0)
Afternoon (from 12:00 to 17:59)	15,259 (31.0)
Evening (from 18:00 to 23:59)	15,500 (31.5)
Night (from 00:00 to 05:59)	6200 (12.6)
Mechanism of injury	
Traffic accident, n (%)	21,166 (43.0)
Fall down, n (%)	14,777 (30.0)
Penetrating injury, n (%)	3448 (7.0)
Others, n (%)	9868 (20.0)
Clinical variables	
Systolic BP, mmHg	112 ± 22.00
Heart rate, bpm	109 ± 26.01
Respiratory rate, /min	13.0± 7.01
Glasgow Coma Scale	14(13–15)
Glasgow Outcome Scale, n (%)	
Death	1118 (2.3)
Vegetative state	520 (1.1)
Severe disability	2960 (6.0)
Moderate disability	6900 (14.0)
Good recovery	37,829 (76.7)
Outcomes	
Admission rate, n (%)	38,428 (78.0)
In-hospital mortality, n (%)	1118 (2.2)
Length of stay in hospital, days	32 (2–60)

Values are presented as mean ± standard deviation (SD) for approximately normally distributed continuous variables, as median (interquartile range, IQR) for skewed continuous variables, and as number (percentage) for categorical variables. Urban indicates metropolitan cities; Rural indicates provinces/non-urban areas. Time of trauma occurrence was categorized as morning (06:00–11:59), afternoon (12:00–17:59), evening (18:00–23:59), and night (00:00–05:59). Systolic BP indicates systolic blood pressure. GCS is reported as median (IQR). GOS categories were assessed at hospital discharge. Admission rate refers to hospital admission following the emergency department visit. In-hospital mortality includes death in the emergency department or during hospitalization. LOS indicates hospital length of stay and is reported as median (IQR). Abbreviations: ED, emergency department; SD, standard deviation; IQR, interquartile range; BP, blood pressure; bpm, beats per minute; GCS, Glasgow Coma Scale; GOS, Glasgow Outcome Scale; LOS, length of stay.

**Table 2:** Comorbidity profile of the study population (*N* = 49,259).

Variables	N(%)
Comorbidity	
No comorbidity, n (%)	16,260 (33)
Comorbidities, n (%)	32,999 (67)
Type of comorbidity	
Congestive heart failure	8629 (26.1)
Cardiac arrhythmia	7657 (23.2)
Psychosis	4247 (12.9)
Renal disease	3142 (9.5)
Hypertension	1402 (4.2)
Peripheral vascular disease	1232 (3.7)
Diabetes mellitus	1021 (3.1)
Valvular disease	899 (2.7)
Pulmonary circulation disorder	851 (2.6)
Chronic pulmonary disease	625 (1.9)
Paralysis	608 (1.8)
Fluid and electrolyte disorders	330 (1.0)
Anemia (blood loss/deficiency)	320 (1.0)
Solid tumor without metastasis	310 (0.9)
Obesity	300 (0.9)
Liver disease	260 (0.8)
Other neurological disorders	210 (0.6)
Peptic ulcer disease	200 (0.6)
Depression	186 (0.6)
Alcohol abuse	180 (0.5)
Hypothyroidism	170 (0.2)
Metastatic cancer	140 (0.4)
Drug abuse	80 (0.2)

Values are presented as number and percentage, and percentages are calculated using the total study population (N = 49,259) as the denominator. Comorbidity was defined based on the Elixhauser comorbidity framework. Patients were classified as having no comorbidity or ≥1 comorbidity. Each comorbidity category was coded as present/absent; therefore, categories are not mutually exclusive, and the sum of percentages across comorbidity types may exceed 100%. Abbreviation: N, number.

**Table 3:** Comorbidity Burden and Clinical Outcomes.

Comorbidity Count	N (%)	Admission (%)	Mortality (%)	LOS, Median (IQR)	Adjusted OR (Mortality)
					(95% CI)
0	16,260 (33.0)	73.3	1.8	28 (2–58)	1 (reference)
1	14,500 (43.9)	76.5	2.1	31 (2–60)	1.29 (1.08–1.55)
2	10,200 (30.9)	79.2	2.6	35 (2–62)	1.55 (1.30–1.85)
≥3	8299 (25.2)	82.8	3.2	39 (3–65)	1.92 (1.58–2.33)
*p* for trend	—	<0.001	<0.001	<0.001	<0.001

Comorbidity counts were categorized into 0, 1, 2, and ≥3 conditions to assess dose–response relationships. Percentages for comorbidity count (1, 2, ≥3) are calculated based on the comorbidity group (n = 32,999), whereas the percentage for 0 comorbidity is based on the total study population (N = 49,259). *p* for trend was calculated using a linear-by-linear association test for categorical outcomes and linear regression for continuous outcomes. Post hoc pairwise comparisons were performed using Bonferroni correction, with the 0 comorbidity group as the reference. Adjusted for age, sex, mechanism of injury, and Injury Severity Score (ISS).

**Table 4:** Individual Elixhauser Comorbidities and Clinical Outcomes.

Comorbidity	N (%)	Admission aOR (95% CI)	Mortality aOR (95% CI)	LOS (B, Days) (95% CI)
Congestive heart failure	8629 (26.1)	1.88 (1.72–2.06)	2.35 (2.10–2.63)	+3.2 (2.8–3.7)
Cardiac arrhythmia	7657 (23.2)	1.21 (1.12–1.30)	1.42 (1.28–1.57)	+1.4 (1.1–1.7)
Valvular disease	899 (2.7)	1.05 (0.89–1.24)	1.18 (0.95–1.46)	+0.5 (−0.1–1.2)
Pulmonary circulation disorder	851 (2.6)	1.56 (1.28–1.89)	1.89 (1.52–2.36)	+2.6 (2.0–3.2)
Peripheral vascular disease	1232 (3.7)	1.12 (1.02–1.24)	1.21 (1.03–1.41)	+0.9 (0.5–1.3)
Hypertension	1402 (4.2)	0.94 (0.86–1.03)	0.98 (0.87–1.11)	+0.1 (−0.2–0.4)
Paralysis	608 (1.8)	1.40 (1.18–1.67)	1.74 (1.38–2.19)	+2.3 (1.7–2.9)
Chronic pulmonary disease	625 (1.9)	1.28 (1.14–1.44)	1.33 (1.12–1.59)	+1.5 (1.0–2.0)
Renal disease	3142 (9.5)	1.90 (1.72–2.10)	2.10 (1.85–2.38)	+2.8 (2.3–3.3)
Diabetes mellitus	1021 (3.1)	1.14 (1.03–1.26)	1.29 (1.12–1.48)	+1.0 (0.6–1.4)
Psychosis	4247 (12.9)	1.09 (1.01–1.18)	1.15 (1.02–1.29)	+0.3 (−0.1–0.8)
Other neurological disorders	210 (0.6)	1.33 (1.14–1.55)	1.47 (1.20–1.80)	+1.6 (1.0–2.1)
Hypothyroidism	170 (0.2)	0.96 (0.81–1.14)	0.91 (0.73–1.14)	−0.2 (−0.7–0.3)
Liver disease	260 (0.8)	2.10 (1.73–2.55)	2.84 (2.32–3.48)	+3.9 (3.1–4.7)
Peptic ulcer disease	200 (0.6)	1.03 (0.87–1.22)	1.05 (0.82–1.34)	+0.4 (−0.2–1.0)
Solid tumor (no metastasis)	310 (0.9)	1.17 (1.02–1.34)	1.33 (1.09–1.63)	+1.2 (0.7–1.7)
Metastatic cancer	140 (0.4)	2.75 (2.30–3.30)	3.95 (3.20–4.88)	+4.2 (3.4–5.1)
Coagulopathy	260 (0.8)	2.01 (1.75–2.32)	2.58 (2.16–3.07)	+3.5 (2.9–4.1)
Obesity	300 (0.9)	1.05 (0.92–1.21)	1.08 (0.90–1.30)	+0.6 (0.2–1.1)
Fluid & electrolyte disorders	330 (1.0)	2.80 (2.50–3.13)	3.12 (2.71–3.58)	+4.6 (4.1–5.1)
Anemia	320 (1.0)	1.26 (1.12–1.42)	1.41 (1.22–1.64)	+1.7 (1.3–2.2)
Alcohol abuse	180 (0.5)	1.18 (1.04–1.34)	1.22 (1.02–1.46)	+0.9 (0.4–1.4)
Drug abuse	80 (0.2)	1.39 (1.08–1.79)	1.51 (1.11–2.05)	+1.3 (0.5–2.1)
Depression	186 (0.6)	1.07 (0.96–1.19)	1.09 (0.92–1.29)	+0.4 (0.0–0.9)

Adjusted for age, sex, mechanism of injury, and Injury Severity Score (ISS). Admission and mortality were analyzed using multivariable logistic regression; length of stay (LOS) using linear regression.

**Table 5:** Comparison of baseline characteristics by comorbidity status among trauma patients.

Characteristics	No Comorbidity (n = 16,260)	Comorbidity (n = 32,999)	*p*-Value
Age, years, median (IQR)	45 (28–62)	61 (47–74)	<0.001
Female	6570 (38.1)	12,527 (42.8)	<0.001
Residential area			
Urban	12,150 (74.8)	21,700 (67.8)	0.01
Rural	4110 (25.2)	11,299 (32.2)	
Clinical variables			
ISS	8 (4–15)	10 (6–19)	<0.001
Outcomes			
Admission rate, n (%)	11,920 (73.3)	25,538 (79.8)	<0.001
In-hospital mortality, n (%)	310 (1.8)	808 (2.5)	<0.001
Length of stay in hospital, days	28 (2–58)	36 (2–62)	0.002
Glasgow Outcome Scale, n (%)			
Death	310 (1.8)	808 (2.5)	<0.001
Vegetative state	140 (0.8)	380 (1.2)	0.02
Severe disability	920 (5.3)	2040 (6.4)	0.01
Moderate disability	2310 (13.4)	4590 (14.3)	0.10
Good recovery	12,580 (78.7)	25,181 (75.6)	0.001
Time of trauma occurrence, n (%)			
Morning (from 06:00 to11:59)	4350 (26.7)	7950 (24.8)	0.65
Afternoon (from 12:00 to 17:59)	4010 (24.8)	10,249 (32.0)	0.02
Evening (from 18:00 to 23:59)	5600 (34.4)	9900 (30.9)	0.04
Night (from 00:00 to 05:59)	2300 (14.1)	4900 (12.2)	0.08
Mechanism of injury, n (%)			
Traffic accident	7000 (43.0)	14,166 (44.3)	0.01
Fall down	5180 (31.8)	9597 (30.0)	0.92
Penetrating injury	1400 (8.6)	3048 (6.4)	0.003
Other blunt (involve unknown injury)	2680 (16.6)	6188 (19.3)	0.02
Clinical variables			
Systolic BP, mmHg	116 ± 21.01	109 ± 23.00	0.002
Heart rate, bpm	112 ± 27	104 ± 25	<0.001
Respiratory rate, /min	13.1 ± 7.8	12.9 ± 6.5	0.219
Glasgow Coma Scale	13 (9–15)	9 (5–14)	0.001
Prehospital characteristics			
Prehospital cardiac arrest, Yes, n (%)	1820 (10.6)	3896 (12.2)	0.001
Call-to-hospital arrival time, min, median (IQR)	32 (22–45)	34 (23–49)	0.04
Method of transport, ambulance (119), n (%)	13,100 (76.0)	25,343 (79.2)	0.002
Method of transport, personal vehicle n (%)	3600 (20.9)	5757 (18.0)	0.001
Method of transport, Helicopter/other, n (%)	530 (3.1)	899 (2.8)	0.20

Values are presented as mean ± standard deviation (SD) for normally distributed continuous variables, as median (interquartile range, IQR) for skewed continuous variables, and as number (percentage) for categorical variables. Comorbidity was defined using the Elixhauser comorbidity framework, and patients were classified into no comorbidity and ≥1 comorbidity groups. ISS indicates Injury Severity Score. Systolic BP indicates systolic blood pressure. GCS indicates Glasgow Coma Scale and is reported as median (IQR). Admission rate refers to hospital admission following the emergency department visit. In-hospital mortality includes death in the emergency department or during hospitalization. Hospital length of stay (LOS) is reported in days as median (IQR). Time of trauma occurrence was categorized as morning (06:00–11:59), afternoon (12:00–17:59), evening (18:00–23:59), and night (00:00–05:59). Prehospital cardiac arrest indicates cardiac arrest before hospital arrival. Call-to-hospital arrival time represents the interval from the emergency call to hospital arrival. Group comparisons used the chi-square test (or Fisher’s exact test when appropriate) for categorical variables and the independent-samples *t*-test or Mann–Whitney U test for continuous variables, as appropriate. All tests were two-sided. Abbreviations: SD, standard deviation; IQR, interquartile range; ISS, Injury Severity Score; BP, blood pressure; bpm, beats per minute; GCS, Glasgow Coma Scale; LOS, length of stay. ISS, Injury Severity Score; 119, Korean emergency medical service system.

**Table 6:** Multivariable regression analysis of admission, mortality, and prolonged LOS.

Variables	Admission (Adjusted OR, 95% CI)	*p*-Value	Mortality (Adjusted OR, 95% CI)	*p*-Value	Prolonged LOS ≥ 7 Days (Adjusted OR, 95% CI)	*p*-Value
Comorbidity (yes vs. no)	1.32 (1.25–1.39)	<0.001	1.41 (1.22–1.63)	<0.001	1.28 (1.21–1.35)	<0.001
Age (per 10 years)	1.22 (1.20–1.35)	<0.001	1.34 (1.22–1.48)	<0.001	1.10 (1.08–1.22)	<0.001
Female (vs. male)	1.08 (1.02–1.14)	0.006	0.91 (0.79–1.05)	0.18	1.05 (0.99–1.11)	0.10
ISS (per unit increase)	1.06 (1.05–1.07)	<0.001	1.08 (1.06–1.10)	<0.001	1.04 (1.03–1.05)	<0.001
Prehospital cardiac arrest	2.15 (1.98–2.33)	<0.001	3.82 (3.21–4.55)	<0.001	1.47 (1.33–1.62)	<0.001

Values are presented as adjusted odds ratios (ORs) with 95% confidence intervals (CIs) derived from multivariable logistic regression models. The models were adjusted for age, sex, injury severity score (ISS), and prehospital cardiac arrest. Hospital admission was defined as admission following emergency department presentation. In-hospital mortality included death occurring in the emergency department or during hospitalization. Prolonged hospitalization was defined as a length of stay (LOS) of ≥7 days. Patients who died before reaching the 7-day threshold were classified as having prolonged hospitalization. Abbreviations: OR, odds ratio; CI, confidence interval; ISS, Injury Severity Score; LOS, length of stay.

## Data Availability

Data used in this study were obtained from the Korea Disease Control and Prevention Agency (KDCA) under an approved application. The data are not publicly available due to third-party restrictions and data use agreements. Access may be granted by KDCA upon reasonable request and subject to the agency’s approval process.

## Abbreviations

The following abbreviations are used in this manuscript:

BP	Blood pressure
bpm	Beats per minute
CI	Confidence interval
ED	Emergency department
EMS	Emergency medical services
GCS	Glasgow Coma Scale
GOS	Glasgow Outcome Scale
ICU	Intensive care unit
IQR	Interquartile range
ISS	Injury Severity Score
LOS	Length of stay
OR	Odds ratio
SD	Standard deviation
